# Lung tumour markers in oncology practice: a study of TPA and CA125

**DOI:** 10.1038/sj.bjc.6600577

**Published:** 2002-11-04

**Authors:** G Buccheri, D Ferrigno

**Affiliations:** Divisione di Pneumologia, Ospedale ‘S. Croce e Carle’, Cuneo, I-12100, Italy

**Keywords:** lung neoplasm, neoplasm staging, prognosis, tissue polypeptide antigen, CA125, tumour markers

## Abstract

Several substances mark the course of lung cancer and may reliably help the clinician in decision-making. This is the first clinical study specifically designed to compare tissue polypeptide antigen and CA 125 tumour associated antigen. Three hundred and eighty-four new lung cancer patients (309 males) were studied at their first clinical presentation and then strictly followed-up. Anthropometric, clinical and laboratory data – including tissue polypeptide antigen and CA 125 tumour associated antigen serum levels – were prospectively recorded. A total of 1000 tissue polypeptide antigen and CA 125 tumour associated antigen serum assays (384 pre-treatment and 616 posttreatment assays) were performed. Both tissue polypeptide antigen and CA 125 tumour associated antigen correlated significantly with the T, N and M stage descriptors at diagnosis (Rho: 0.200, 0.203, 0.263 and 0.181, 0.240, 0.276, respectively), and then with the objective response to treatment (Rho: 0.388 and 0.207, respectively). A pleural neoplastic involvement was mainly associated to an increase of CA 125 tumour associated antigen (Rho: 0.397). Both tissue polypeptide antigen and CA 125 tumour associated antigen were strongly predictive of the patients' outcome, as assessed by the univariate analysis of survival (log-rank test: 37.24 and 29.01) and several Cox' proportional hazards regression models. The two marker tests are similarly helpful and appear complementary, given the low inter-marker correlation and their independent prognostic capability.

*British Journal of Cancer* (2002) **87**, 1112–1118. doi:10.1038/sj.bjc.6600577 www.bjcancer.com

© 2002 Cancer Research UK

## 

Tumour markers are not only of significance to the researcher in understanding tumour biology, but also to the clinician in treating patients with cancer (Pamies and Crawford, 1996). In oncology practice, tumour markers may be helpful in the diagnosis and in the pathologic classification. Plasma levels of serum tumour markers may reflect both stage of disease and prognosis. When measured serially after a diagnosis is established, they may aid in assessing the response to treatment, in monitoring the spontaneous course of the illness, and in surveillance for tumour recurrences (Coombes and Powels, 1982).

Also in lung cancer, the expression of serum biomarkers is various and abundant (Buccheri, 1999). Among them, markers originating from the cytoskeleton are of conceptual and practical interest (Buccheri and Ferrigno, 2001a). Tissue Polypeptide Antigen (TPA) is identified as a degradation product of the cytoskeleton, formed by the cytokeratins 8, 18 and 19 (Bjorklund, 1978). CA 125 tumour-associated antigen (CA125) is another interesting lung tumour marker. It is a membrane glycoprotein of the serous ovarian cancer cell line OVCA 433 recognised by the monoclonal antibody OC125 (Bast *et al*, 1981). The use of CA125 for diagnosis and follow-up of ovarian cancer was soon well defined (Bast and Klul, 1983). Currently, it is clear that CA125 might also be used as a marker of other cancers, including lung cancer (Diez *et al*, 1991).

In this study, we describe the results of a new Cuneo Lung Cancer Study Group's (CuLCaSG) study aimed to compare CA125 and TPA in each of their most important clinical applications. In particular, we compared the two marker tests in: (1) Disease extent evaluation at diagnosis; (2) Treatment response follow-up assessment; and (3) Prognostication. To our knowledge, this is the first report of this type.

## PATIENTS AND METHODS

### Patients' database and study design

In 1982, a group of chest physicians, who later became known as CuLCaSG, started working in the field of lung cancer. The group is still active at the Pulmonary Unit of the ‘S. Croce e Carle’ hospital, in the city of Cuneo, Piedmont, Italy. The hospital serves the whole Cuneo Province as Third Referral Institution. Among the prime actions of the CuLCaSG, a database for patients with carcinoma of the lung, effective in January 1983, was created. All lung cancer patients, referred to a physician of the group, were managed uniformly and their clinical data recorded on computer.

All patients, seen in 1997 and afterwards, were included in this study if they had a pathologic diagnosis of NSCLC (World Health Organization, 1991). Based on our study requirements, all patients had undergone each of the following: (1) Complete and accurate assessment of the extent of disease; (2) Routine tumour marker assays, including the determination of both pre-treatment and post-treatment TPA and CA125 plasma levels; (3) A strict follow-up and a yearly check of their status (for the few cases who abandoned their follow-up programs). Follow-up re-evaluations consisted of clinical, laboratory, and radiological reassessments performed at 3-week intervals during chemotherapy, and every 3–6 weeks in case of palliative radiotherapy, or no anti-cancer treatment. Patients treated with radical surgery were scheduled for follow-up visits at longer intervals, ranging 3–6 months. Based on our previous experience (Buccheri, 1999), tumour marker assay was considered an essential part of the patients' clinical evaluation and no formal informed consent was required for this study.

Survivals were recorded from the time of histological diagnosis to death, or to the last clinical examination or telephone contact with the patient himself, the family, the house doctor, or the municipal office of the registry. Consequently, both the duration of survival and the status of dead or alive at the closure of the study (i.e., at the end of December 2001) was available for all patients. As at December 2001, 176 patients (46%) were still alive, after a median follow-up time of 23 weeks.

During the 5 year-period of study, 384 new eligible patients were seen and included into the study. In all, 1000 twin TPA and CA125 plasma measurements (616 during or after treatment) were obtained. The anthropometric and clinical characteristics of the study cohort are shown in Table 1

**Table 1:**
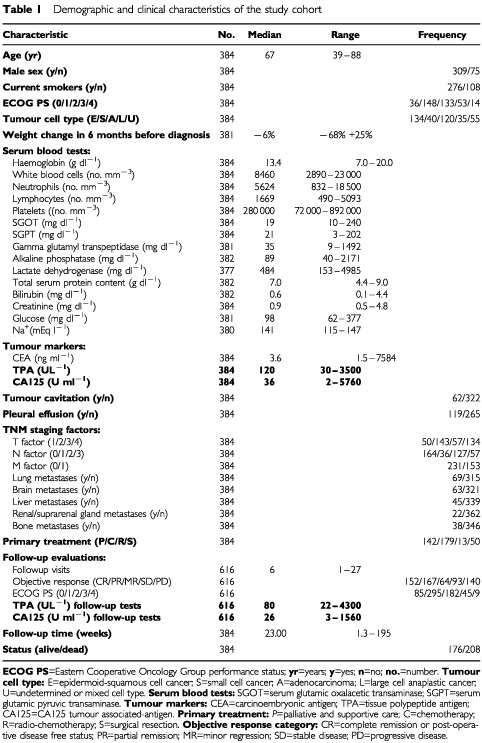
Demographic and clinical characteristics of the study cohort

.

### CA125 and TPA assays

Sera for CA125 and TPA were stored at −20°C, a temperature that satisfactorily ensures the stability of blood specimens, and assayed three times per week in the central laboratory of the ‘S. Croce and Carle’ hospital. The laboratory is located in the ‘S. Croce’ building. It receives blood samples from many medical and surgical wards, including the lung unit of the ‘A. Carle’ building. Since we provide no clinical information, biologists have no means of knowing even the disease for which a particular test is required.

Plasma measurements were performed following the manufacturer's instruction and using the reagents contained in the commercial kits of AB Sangtec Medical Co., Bromma, Sweden (TPA) and B-R-A-H-M-S Diagnostica GmbH, Berlin, Germany (CA125). Reference values were up to 80 U l^−1^ (TPA) and 35 U ml^−1^ (CA125).

### Other pre-treatment evaluations

All patients included in this report were studied with a computed tomography (CT) of thorax, upper abdomen and brain. Mediastinal nodes were labelled as abnormal if they were 1.5 cm or larger (smallest diameter). All CT scans were interpreted with no restriction to the clinical information available at the time of the exam. In addition to CT scanning, the baseline clinical evaluation included physical examination, routine lab tests, bronchoscopy, and functional respiratory tests. In nearly half of the sample, the baseline work-up was supplemented by a technetium-99m methylene diphosphonate bone scan. Other imaging tests (including X-rays, CT and magnetic resonance imaging of the skeleton, ultrasonographic studies of the abdomen, and other organ-specific tests) were optional and performed as clinically indicated. The pre-treatment staging evaluation was pathological in 50 patients who underwent surgical resection of the tumour (13% of the cohort). All diagnostic and staging tests were obtained within a 2–3-week period.

### Data analysis and statistical considerations

Data were statistically analysed using the SPSS package for Windows, Version 9.0 (SPSS Inc., Chicago, IL, USA). Non-parametric tests (Siegel, 1956) were used to test relationships and differences among plasma levels of tumour markers or between these factors and other variables. In particular, the Spearman rank test, the Kruskall–Wallis test, and the median tests were used as appropriate. Medians and ranges described continuous variables because for many variables, including TPA and CA125, their distribution was not Gaussian (Siegel, 1956).

Diagnostic capabilities were intended, for both TPA and CA125, to show the presence (or absence) of a specific disease's condition, such as a metastatic spread or an objective response to treatment. To assess diagnostic capabilities, we used the receiver-operating characteristic (ROC) curves (Mcneil *et al*, 1975), whose circumscribed areas (the area under the curve) give an estimate of the diagnostic efficiency (Hanley and Mcneil, 1982).

All the parameters assessed at diagnosis and listed in Table 1 were considered for survival analysis. Survival time was the dependent variable. Survival curves were assessed according to the Kaplan–Meier method (Kaplan and Meier, 1958), and plotted at weekly intervals. Differences among curves were assessed according to the log-rank test (Peto *et al*, 1977). To control for the effect of potential confounders, a multivariate analysis, based on Cox's proportional hazards regression model (Cox, 1972), was performed. For each variable included in the model the proportional hazard assumption was tested graphically.

A probability (*P*) level <0.05 was considered statistically significant. All statistical tests were two-sided.

## RESULTS

### Inter-marker correlation and other clinical relationships

**Figure 1:**
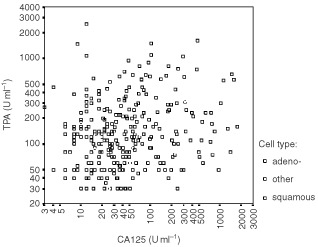
Scatter-plot showing pre-treatment levels of CA125 and TPA, measured blindly of clinical information, in 384 new lung cancer patients (Spearman Rho Correlation Coefficient: 0.24).

The plasmatic levels of the two markers, measured at the patients' first clinical presentation, are plotted in Figure 1

. In the graph, each main cell type is graphically distinct with open boxes of different grey tonality. The Spearman rank correlation index was rather weak (Rho: 0.24), but still significant given the elevated number of measurements (*P*<0.001). This correlation indicates that there is a certain, though modest, overlap in the expression of the two markers. As expected, the correlation between CA125 and another more biologically similar tumour marker, Carcinoembryonic Antigen (CEA), was remarkably higher (Rho=0.34). Both CA125 and TPA were significantly correlated with the serum levels of lactate dehydrogenase, with the T, N, and M classifications, the presence of liver metastasis, the scale of efficacy in the adopted treatment, the objective response to treatment, the posttreatment status of disease and the performance status measured during the follow-up (Table 2

**Table 2:**
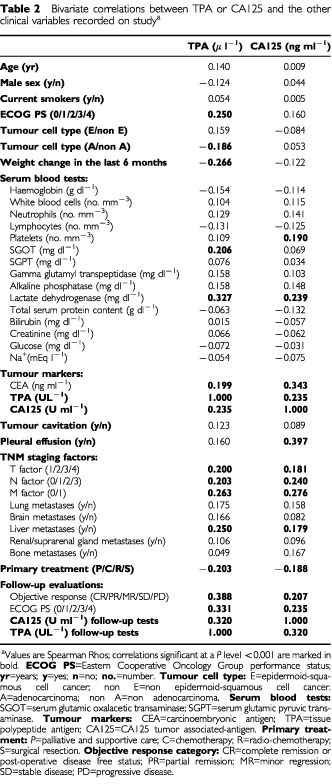
Bivariate correlations between TPA or CA125 and the other clinical variables recorded on study^a^

). In addition, TPA was associated or more closely associated with other clinical factors, such as the amount of weight loss, or with a few other lab tests, such as liver enzymes and blood cell counts (Table 2). Typically, CA125 exhibited a strong association with the presence of pleural fluid (Rho=0.40). Exploiting such a characteristic, CA125 may be used to predict a serous membrane involvement, normally a pleural metastatic spread of the disease (Table 3

**Table 3:**
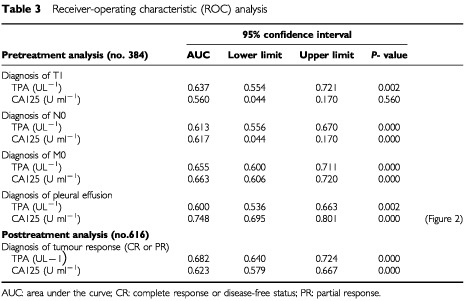
Receiver-operating characteristic (ROC) analysis

and Figure 2

**Figure 2:**
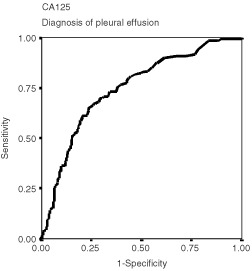
Receiver-operating characteristic (ROC) curves showing the capability of CA125 to diagnose a cancerous pleural effusion (area under the curve: 0.748, 95% confidence interval: 0.695–0.801, *P*<0.001).

). Using the CA125 normality reference, we could calculate for CA125 a sensitivity rate of 77% and a specificity rate of 60% in the diagnosis of pleural neoplastic effusion.

### Diagnosis of important clinical conditions

Table 3 shows the results of multiple ROC analyses intended to assess the different diagnostic capability of the two markers under evaluation. In particular, the capability to recognise three important clinical conditions was considered. We analysed the diagnosis of limited disease, as evidenced by: (1) a T1 category of tumour involvement; (2) a N0 category of lymph node involvement; and (3) a M0 category of metastatic disease. In addition, the posttreatment diagnosis of objective response to treatment was considered. All these analyses, except the CA125 ability to recognise a T1 disease, resulted in high statistical significance with a substantially similar behaviour between the two tests (Table 3).

### Survival studies

Figures 3

**Figure 3:**
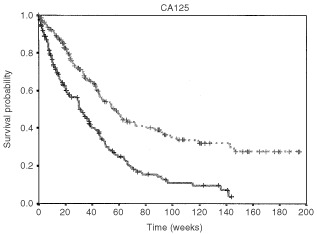
Kaplan–Meier's estimates of the survival function based on the 50th percentile of the CA125 distribution (dashed line: values below the median; values equal to or above the median: continuous line; log-rank test: 29.01, *P*<0.001).

and 4

**Figure 4:**
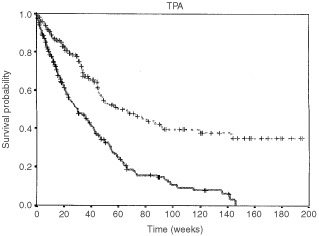
Kaplan–Meier's estimates of the survival function based on the 50th percentile of the TPA distribution (dashed line: values below the median; values equal to or above the median: continuous line; log-rank test: 37.24, *P*<0.001).

show the Kaplan–Meier analyses of the survival, based on the distribution of the plasma levels of CA125 (Figure 3) and TPA (Figure 4): categorisation was made using the median as the cut-off value. Both graphs clearly show that a low marker level was strongly associated with a more favourable outcome (log-rank test: 29.01 and 37.24 for CA125 and TPA, respectively; *P* always <0.001).

Table 4

**Table 4:**
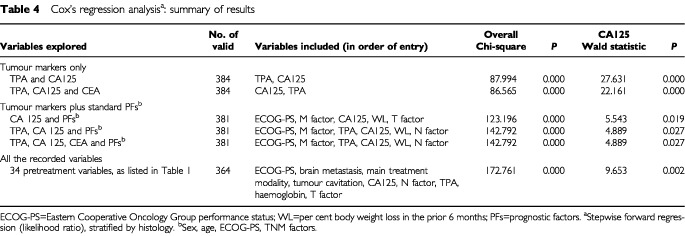
Cox's regression analysis^a^: summary of results

summarises the best multivariate models obtained, whether they included only tumour markers, or CA125 and/or TPA along with a short list of standard prognostic factors (i.e., sex, age, performance status, and the TNM factors), or, finally, whether they included all the variables listed in Table 1. In short, while CEA possesses prognostic information that is contained within either TPA or CA125, both these latter substances are independent prognostic predictors. They remain independent cofactors when standard prognostic factors are taken into account and even when a quite large miscellany of 33 lab and clinical variables are considered.

## DISCUSSION

Nearly half a century ago, B Bjorklund (1953) was first to propose TPA as possible marker for epithelial tumours. He obtained the antigen that called Tissue Polypeptide Antigen (TPA), mixing different tumours and producing an immune serum against the mixture. TPA is currently identified as a degradation product of the cytoskeleton, formed by the cytokeratins 8, 18 and 19 (Bjorklund, 1978). It is expressed and immunohistochemically detectable in the cytoplasm of epithelial tumour cells, including bronchial cancer cells (Bjorklund, 1980).

Between 1986 and 1987, our group reported two clinical studies which aimed to evaluate diverse serum tumour markers of lung cancer (Buccheri *et al*, 1986, 1987). We found TPA the most useful one, even more useful than CEA (Buccheri *et al*, 1987). We continued measuring TPA in all our patients with lung cancer and this allowed us to re-evaluate and periodically confirm earlier findings (Buccheri and Ferrigno, 1988, 1992, 1995a,b; Ferrigno *et al*, 1989; Buccheri *et al*, 1993). Recently, the clinical yield of TPA was determined either in the pre-treatment assessment of operability or in the follow-up evaluation of the status of disease. Using appropriate thresholds, surgical resectability (Buccheri and Ferrigno, 1995b, 2001b) and objective responses to treatment (Buccheri and Ferrigno, 1995a) could be predicted with an accuracy similar to that of computed tomography.

Evidence concerning the clinical value of CA125 is also remarkable and, again, it is mainly due to one group of investigators (Diez *et al*, 1991, 1994, 1996; Picardo *et al*, 1994). Like TPA, the concentration of CA125 in the sera or in tumour cell cytosols of lung cancer patients has been found increased in the most advanced stages of disease (Diez *et al*, 1991; Kimura *et al*, 1990; Picardo *et al*, 1994) and prognostically significant (Diez *et al*, 1994, 1996).

The current study was designed to compare, in a comprehensive manner and in a sufficiently large population, the above-discussed two tumour markers, i.e. CA125 and TPA. To our knowledge, there are no prior studies that have compared CA125 and TPA in lung cancer.

Two prior investigations had reported the clinical yield of a panel of lung tumour markers, including CA125 and the Cytokeratin 19-Fragments (Cyfra 21-1), an analogue of TPA (Buccheri and Ferrigno, 2001a). To assess their diagnostic capability, 189 patients with primary lung cancer and 50 healthy subjects were studied by Molina *et al* (1994). Abnormal Cyfra 21-1 and CA125 values were found in 53.6% and 39%, respectively, of the patients with active cancer (Molina *et al*, 1994). More recently, Ando *et al* (2001) investigated the same two tumour markers, along with other marker substances, in 312 patients (200 patients with adenocarcinoma; 112 patients with squamous cell carcinoma). In adenocarcinoma patients, CEA showed the highest positivity rate (46.5%), followed by CA125, whose positivity rate increased with the stage of disease. In squamous cell carcinoma patients, the positivity rate of Cyfra 21-1 (48.2%) was the second highest, but increased as the stage advanced.

The current study investigated the correlation between disease extent and CA125, but also took into consideration two other important clinical applications, i.e., the assessment of the response to treatment – or the evaluation of the post-treatment status of disease – and the prediction of outcome. Our results show that both TPA and CA125 are similarly effective in each of the above-mentioned applications with few, little differences. Both TPA and CA125 correlated well with the extent of disease (Rho=0.263 and 0.276, respectively), and the response to treatment (Rho: 0.241 and 0.238). Univariate analyses of survival showed that abnormally elevated values of both CA125 and TPA were strongly associated with the worst prognosis (*P*=0.001) and, finally, Cox's multivariate models confirmed their prognostic significance, demonstrating their independence from any other possible survival determinant. The most important difference between TPA and CA125, was the CA125 specific response to the presence of pleural effusions. This is in accordance with findings from another report (Kimura *et al*, 1990) and may be explained by the fact that CA125 is an antigen that normally exists in the ectodermal cells of peritoneum and pleura (Kabawat *et al*, 1983).

## CONCLUSIONS

For a biologic parameter to be used as a marker test, it is essential that the assay is cheap, simple, objective, comparable, reproducible and that the result is available in a short space of time to the doctor. In our opinion, the serum quantification of TPA and CA125 clearly meets these requirements. We encourage clinicians treating lung cancer patients to assay them before treatment (to exploit their capability to give an insight into the severity of the illness and into its possible outcome) and, serially, during and after treatment (to help in deciding the status of the disease and its response to the treatment). CEA appears less useful than CA125, since its informative content appears, in some way, shaded by CA125, and could be omitted.
